# Three-Year Trends of Healthcare-Associated Infections and Antibiotic Use in Acute Care Hospitals: Findings from 2016–2018 Point Prevalence Surveys in Sicily, Italy

**DOI:** 10.3390/antibiotics10010001

**Published:** 2020-12-22

**Authors:** Martina Barchitta, Andrea Maugeri, Maria Clara La Rosa, Claudia La Mastra, Giuseppe Murolo, Antonella Agodi

**Affiliations:** 1 Department of Medical and Surgical Sciences and Advanced Technologies “GF Ingrassia”, University of Catania, 95123 Catania, Italy; martina.barchitta@unict.it (M.B.); andrea.maugeri@unict.it (A.M.); mariaclara.larosa@unict.it (M.C.L.R.); claudia.lamastra@unict.it (C.L.M.); 2 Regional Health Authority of the Sicilian Region, 90145 Palermo, Italy; giuseppe.murolo@regione.sicilia.it; 3 AOU Policlinico “G. Rodolico-San Marco”, 95123 Catania, Italy

**Keywords:** healthcare-associated infections, antibiotic use, antimicrobial agents, acute care hospitals, point prevalence survey, public health

## Abstract

Repeated point prevalence surveys (PPSs) of healthcare-associated infections (HAIs) and antibiotic use are crucial to monitor trends over years at regional level, especially in countries with decentralized healthcare systems. Here, we reported the results of three PPSs conducted in Sicilian acute care hospitals (Italy) from 2016 to 2018, according to the European Centre for Disease Prevention and Control protocol. Overall, prevalence of patients with at least one HAI was 5.1% in 2016, 4.7% in 2017, and 5.1% in 2018, without a significant trend over years (*p* = 0.434). At the patient level, the most important factor associated with HAIs was antibiotic use, since patients receiving at least one antimicrobial were more likely to be infected than those who did not receive antimicrobials (OR = 18.87; 95%CI = 13.08–27.22). The analysis of the prevalence of antibiotic use indicated a significant trend across years of the PPSs: 50.5% of patients received at least one antimicrobial agent in 2016, 55.2% in 2017, and 53.7% in 2018 (*p* < 0.001). The most common indication for antimicrobial prescription was medical prophylaxis, while third-generation cephalosporins represented the most frequently used class of antimicrobial agents, followed by fluoroquinolones and combinations of penicillins. Our study confirms that HAIs still remain a major public health issue, which could be intensified by antibiotic abuse. This raises the need for infection prevention and control and antibiotic stewardship programs aimed to improve knowledge about appropriate antibiotic prescription and to reduce the use of broad-spectrum antimicrobials.

## 1. Introduction

Healthcare-associated infections (HAIs) are one of the major threats for public health worldwide, due to their significant impact on mortality, hospital stays, and assistance costs [1,2,3,4,5,6]. In 2011–2012, the European Centre for Disease Prevention and Control (ECDC) coordinated the first point prevalence survey (PPS)—which was then repeated in 2016–2017—to collect data on HAIs and antimicrobial use in acute care hospitals at the European level [7,8]. The ECDC’s PPSs—confirming the importance of HAI surveillance for infection prevention and control (IPC) programs—added to previous studies and reviews [9,10,11,12,13,14]. In general, prevalence studies certainly have intrinsic limitations, but on the other hand, require much less resources than incidence studies to estimate the burden of HAIs and the impact of Infection Prevention and Control (IPC) programs at the national level [15,16]. Indeed, the ECDC’s PPS still represents the largest survey of its kind performed in Europe, with more than 1000 hospitals in 33 countries. In particular, estimates of the ECDC indicated HAIs as a major public health issue, with ~6% of all patients infected with at least one HAI on a given day [7,8]. Repeating the survey at predefined intervals could be a useful way to avoid some of its limitations [16,17] and to evaluate trends of HAIs and antimicrobial use across years. This is especially important in countries with decentralized healthcare systems, such as Italy, where regional administrations play a crucial role in IPC programs [18,19,20,21,22]. Here, we reported the results of three PPSs conducted in Sicilian acute care hospitals (southern Italy) from 2016 to 2018. We first analyzed changes in the overall prevalence of HAIs and for specific types of infections. Next, we analyzed which patients’ factors (i.e., demographics, clinical severity, presence of invasive devices, and antimicrobial treatment) were associated with the prevalence of HAI. Finally, we described prevalence of antimicrobial use, the main indications for treatment, and the most used antimicrobial agents across different PPSs.

## 2. Results

### 2.1. Characteristics of Patients

The present study used data from 18,852 patients surveyed during the three PPSs in Sicilian acute care hospitals. Specifically, patients involved in each survey were 6448 in 2016, 5974 in 2017, and 6430 in 2018. Table 1 summarizes the main characteristics of patients stratified by the year of the survey. Overall, the mean age was 59.9 years (standard deviation, SD = 24.2), but it significantly increased from 2016 (mean = 58.6; SD = 24.7) to 2018 (mean = 62.4; SD = 22.2; *p* < 0.001). In line with ageing populations, we also noted an increasing percentage of surgical interventions (*p* < 0.001), a higher McCabe severity of illness score (*p* < 0.001), and a higher probability to be exposed to invasive devices (*p*-values < 0.001) from 2016 to 2018.

### 2.2. Prevalence of Healthcare-Associated Infections

**Figure 1:**
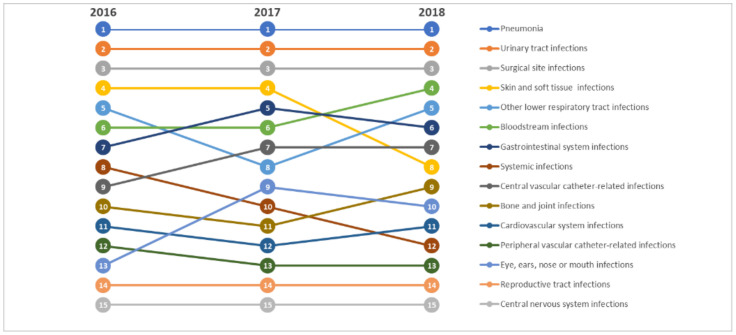
Ranking of types of HAI by year of the Point Prevalence Survey.

**Figure 2:**
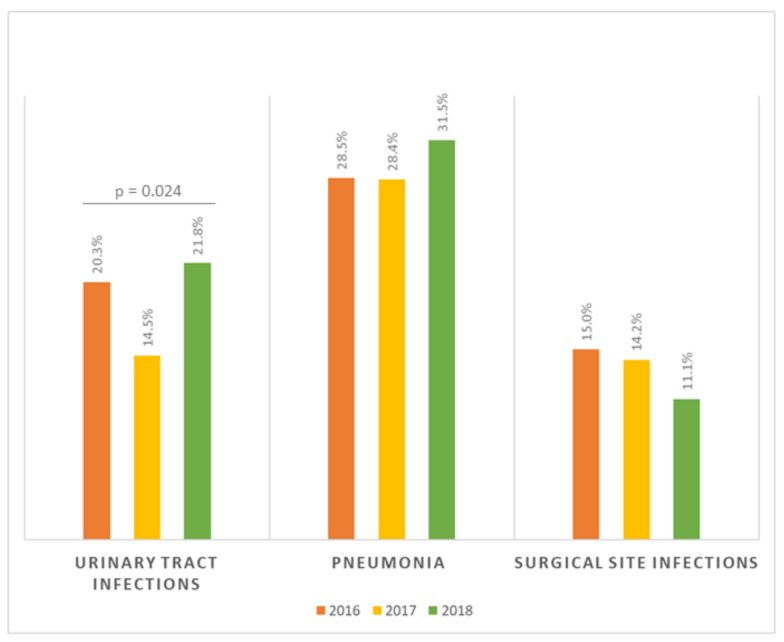
Prevalence of the three most common HAIs by year of the Point Prevalence Survey.

Overall, the number of HAIs was 354 in 2016, 317 in 2017, and 371 in 2018. Instead, the prevalence of patients with at least one active HAI on the survey day was 5.1% in 2016, 4.7% in 2017, and again 5.1% in 2018, without a significant trend across years (*p* = 0.434). From a total of 1037 HAIs for which information was available, the most frequently reported types were pneumonia (29.6%), urinary tract infections (19.1%), and surgical site infections (13.3%), with no significant changes in the top-three ranking from 2016 to 2018 (Figure 1). By contrast, bloodstream infections and those of sensory organs were rising (from 6th to 4th and from 13th to 10th, respectively), while systemic infections and those of skin and soft tissues were declining (from 8th to 12th and from 4th to 8th place, respectively) (Figure 1). The distribution of HAI types for each year of the PPS is reported in the Supplementary Materials Table S1. In particular, we observed a significant trend in urinary tract infections, which accounted for 20.3% of all HAIs in 2016, 14.5% in 2017, and 21.8% in 2018 (*p* = 0.024). No significant trends were evident for pneumonia and surgical site infections (Figure 2).

### 2.3. Factors Related to Healthcare-Associated Infections

In Table 2, we next compared the main characteristics between patients with an active HAI on survey day (i.e., infected patients) and those who did not have active HAI (i.e., non-infected patients). The univariate analysis showed that the infected group was older (*p* < 0.001) and with a higher proportion of patients who underwent surgery (*p* < 0.001), who received fatal or rapidly fatal diagnoses (*p* < 0.001), and who were exposed to invasive devices (*p*-values < 0.001 except for peripheral venous catheter). We also observed an extremely higher prevalence of antibiotic use in infected patients than in their non-infected counterparts (*p* < 0.001). The logistic regression analysis confirmed much of these findings. In fact, patients with a fatal or a rapidly fatal diagnosis were more likely to be infected (odds ratio, OR = 1.70; 95% confidence interval, 95%CI = 1.39–2.07 and OR = 1.91; 95%CI = 1.57–2.31, respectively), as well as those who were exposed to intubation or central venous catheter (OR = 1.88; 95%CI = 1.49–2.38 and OR = 3.21; 95%CI = 2.61–3.95, respectively). However, the most important factor associated with the infected status was the prevalence of antibiotic use, so much that patients receiving at least one antimicrobial were approximately 19 times more likely to be infected than those who did not receive antimicrobials (OR = 18.87; 95%CI = 13.08–27.22).

### 2.4. Prevalence of Antibiotic Use, Indication, and Antimicrobial Agents

**Figure 3:**
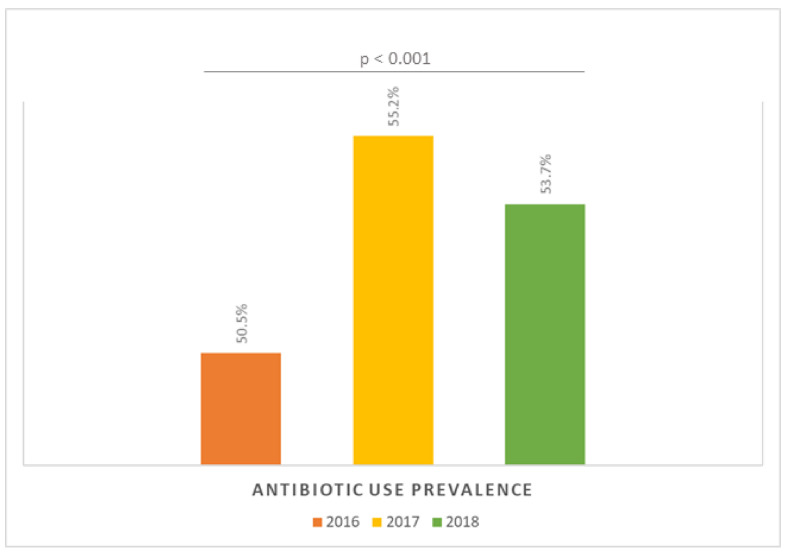
Prevalence of antibiotic use by year of the Point Prevalence Survey.

The analysis of the prevalence of antibiotic use pointed out a significant trend across years of the PPS (Figure 3). Particularly, 50.5% of patients received at least one antimicrobial agent in 2016, while this proportion increased to 55.2% in 2017 and then slightly decreased to 53.7% in 2018 (*p* < 0.001). However, there was no significant correlation between prevalence of HAI and that of antimicrobial use (*p* = 0.634). By contrast, we noted a slight but significant correlation between the number of patients surveyed and prevalence of HAI (r = 0.226; *p* = 0.003; Supplementary Materials Figure S1).

The indication for antimicrobial use was documented in patients’ medical records for 12,841 prescriptions (Table 3). In general, antimicrobials were most frequently prescribed for medical prophylaxis, but we noted some variations for other indications across years. Indeed, surgical prophylaxis decreased from 2016 to 2018 (*p* < 0.001), while treatment of a community-acquired infection increased (*p* = 0.017). A significant trend was also observed for the treatment of a hospital infection, which was characterized by a negative peak in 2017 (*p* < 0.001).

**Figure 4:**
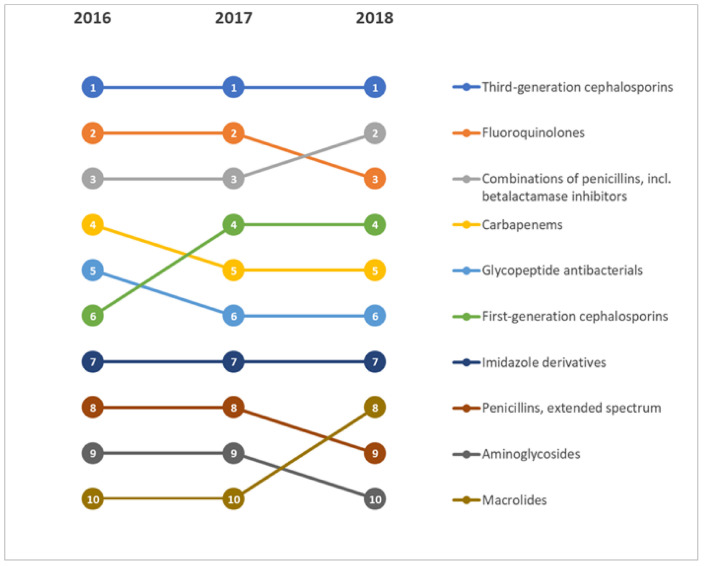
Ranking of antimicrobial agents by year of the Point Prevalence Survey.

From 2016 to 2018, third-generation cephalosporins represented the most frequently used class of antimicrobial agents, followed by fluoroquinolones and combinations of penicillins. In particular, we observed a decreasing trend of fluoroquinolones use from 2016 to 2018 (*p* < 0.001; Figure 4). Overall, the distribution of antimicrobial agents for each year of the PPS is reported in the Supplementary Materials Table S2. From 2016 to 2018, we also noted a decreasing trend for the use of glycopeptide antibacterials and an increasing trend for first-generation cephalosporins and macrolides.

## 3. Discussion

The World Health Organization states that surveillance is one of the core components for effective IPC programs against HAIs [14], setting out the framework within which HAIs occur and reinforcing the attention of hospitals and healthcare professionals at different levels. Repeating the surveys at fixed intervals, moreover, helps to describe trends of HAIs over the years and to identify new needs. Here, we reported the prevalence of HAIs and antibiotic use in Sicilian acute care hospitals from 2016 to 2018, using data collected through the ECDC PPS protocol. In fact, measuring and collecting data through standardized protocols makes it possible to provide a benchmark and to analyze trends within and between hospitals [7,23].

In Sicilian acute care hospitals, the prevalence of HAIs tended to settle around 5%, an estimate that was lower than 8% reported by the 2016–2017 Italian PPS and ~6% found in the 2011–2012 and 2016–2017 European PPSs [7,8,24]. Prevalence of HAIs in Sicilian hospitals was also slightly lower than estimates reported by Arnoldo and colleagues for the Friuli Venezia Giulia region in northern Italy [25]. Notably, the most frequent HAIs were pneumonia, urinary tract infections, and surgical site infections, a scenario that was in line with those reported in Europe and Italy [7,8,24,25]. Unlike Arnoldo and colleagues, however, we did not find a significant trend in the prevalence of HAIs over the years. Data from Friuli Venezia Giulia, indeed, showed that prevalence of HAIs decreased from 7.1% in 2011 to 5.8% in 2017 [25]. By contrast, our analysis demonstrated that prevalence of HAIs remained stable from 2016 to 2018, with a negative but not significant peak in 2017. This was partly comforting since patients participating in the Sicilian PPSs became older and more severe over the years, although it is well known that aging and disease severity are risk factors for HAIs [26,27,28]. In line, we noted an increasing trend in the presence of invasive devices from 2016 to 2018, but it also did not result in an increased prevalence of HAIs. It is worth mentioning that results from logistic regression indicated disease severity (i.e., assessed using the McCabe score) and the presence of invasive devices as factors associated with the prevalence of HAIs. Thus, this proves the emerging need for identifying patients at higher risk of HAIs at an early stage [27,28] and for improving the management of invasive devices and surgical procedures [16,29,30,31,32,33].

Yet, at the patient level, the main factor associated with HAIs was antibiotic use, even if the cross-sectional nature of PPSs did not allow an understanding of their causal relationship. Indeed, it was not clear whether antibiotic use was a cause or a consequence of HAIs. Actually, at the hospital level, we failed in demonstrating a correlation between prevalence of antibiotic use and prevalence of HAIs. Thus, our analysis did not highlight higher prevalence of HAIs in hospitals with higher prevalence of antimicrobial use, something that was instead observed in the 2012 European PPS, and that might also reflect some difficulties in confirming the case definition for those infections with undocumented signs and symptoms. In fact, the good correlation reported by the European analysis suggested that PPS staff probably often followed the prescribers’ subjective opinion to define HAIs, instead of applying criteria of the EDC PPS protocol [7].

Despite the growing impact of antibiotic abuse on HAI risk—especially for infections caused by multidrug-resistant bacteria [34,35,36,37,38,39]—the proportion of patients receiving at least one antimicrobial agent in Sicily increased from 50.5% in 2016 to 53.7% in 2018. Overall, the first reason for prescribing antibiotics was medical prophylaxis, ranging from 34.1% in 2016 to 36.4% in 2018. A proportion that was much higher than those in Europe (11% in 2011–2012 and 10% in 2016–2017), Italy (23.3%), and Friuli Venezia Giulia (19%), where the first reason for prescribing antibiotics was the treatment of community infections [7,24,25,40]. In general, medical prophylaxis referred to the use of antimicrobials for the general purpose of preventing infections, but a limited number of indications for this kind of prophylaxis are reported in relevant guidelines [40]. For this reason, the high level of antimicrobials given for medical prophylaxis might hide a proportion of prescriptions without clear indication and, therefore, unnecessary. With respect to antimicrobial agents, findings were in line with those reported previously [7,24,25,40]: even in Sicily, third-generation cephalosporins, fluoroquinolones, and combinations of penicillins represented the most frequently used classes, with slight variation over the years. These findings denoted the need for improving antibiotic prescribing and for rationalizing the use of broad-spectrum antimicrobials in Sicilian acute care hospitals.

Our work has some limitations that should be considered when interpreting results and that are common to all the PPS. First, prevalence indicators and their comparison over the years might be affected by the number and type of participating hospitals. Second, differences in data validity and case ascertainment might influence the prevalence of HAIs and antibiotic use per hospital, but the regional averages could be considered more valid since underreporting and overreporting could be balanced. Third, information on type HAI, indication for treatment, and antimicrobial agent used were not always available for all the participants.

## 4. Materials and Methods

In 2016, in the framework of a Regional Action Plan on prevention of HAIs, AMR, and inappropriate use of antimicrobials [41], the Sicilian Health Authority launched the first regional PPS of HAIs and antimicrobial use in acute care hospitals, which was followed by two further editions in 2017 and 2018 [42]. Specifically, the Sicilian PPSs adopted the 5.1 version of the ECDC protocol and definitions used for the European PPS [43]. All acute care hospitals regardless of their size—and specifically all wards included in acute care facilities—were eligible for inclusion, except accident and emergency departments. In line with the ECDC protocol [43], each ward carried out a single-day surveillance, including all patients admitted to the ward before or at 8 a.m. and not discharged from the ward at the time of the survey. For each ward, data were referred to the single day of the survey unless otherwise indicated, while the total time frame for data collection for all wards of a single hospital did not exceed three weeks. In particular, 85 acute care hospitals participated in the 2016 PPS, 70 in 2017 PPS, and 69 in 2018 PPS. Prevalence of HAIs was computed as the number of patients with an active HAI on survey day divided by the total number of surveyed patients. The definition of an active HAI relied on the presence of signs and symptoms, the day of onset of symptoms in relation to hospital admission, and the possible presence of an invasive device before infection. However, the full list of criteria, also in relation to specific cases, is reported in in the ECDC protocol [43]. Prevalence of antibiotic use was computed as the number of patients receiving at least one antimicrobial at the time of the survey divided by the total number of patients. In general, given or planned administration of antimicrobials was registered at the time of the survey only, except for antimicrobials for surgical prophylaxis that were registered if given the day before the survey. All the criteria for registering indications for treatment are described in the ECDC protocol [43]. It is worth mentioning that data on HAIs and antimicrobial use were collected separately, with no intention to discuss about the appropriateness of prescription. Indeed, the list of indications for antimicrobial use proposed by the ECDC protocol referred to treatment intention of an infection [43].

All data collected at the hospital and patient level were managed confidentially and anonymously during statistical analysis, which was performed using SPSS software (version 26). Patients’ characteristics, prevalence of HAIs and antibiotic use, and indications for treatment were compared across years of PPS using the Chi-squared test or the Analysis of Variance (ANOVA). We also compared patients’ characteristics between infected and non-infected individuals using the Student’s t-test or the Chi-squared test. Accordingly, results were reported as frequency and percentage or mean and SD. We further applied a logistic regression analysis to assess factors that were mainly associated with HAIs. Results were reported as ORs with their 95%CIs. A *p*-value < 0.05 was considered as statistically significant for all the analyses.

## 5. Conclusions

In conclusion, this is the first study examining the trend of HAIs and antibiotic use in Sicily, using data from repeated PPSs. In particular, we confirmed that HAIs still remain a major public health issue, which could be aggravated by antibiotic abuse. This raises the need for IPC and antibiotic stewardship programs aiming to improve knowledge about antibiotic prescription and to reduce the use of broad-spectrum antimicrobials. Organizing and carrying out constant HAI surveillance definitely enhances the awareness of healthcare workers at each level, but considerable efforts to harmonize the interpretation of definitions are still needed.

## Figures and Tables

**Table 1:** Characteristics of patients by year of the Point Prevalence Survey.

Characteristics	2016 (n = 6448)	2017 (n = 5974)	2018 (n = 6430)	*p*-Value ^a^
**Age, years ^b^**	58.6 (24.7)	58.5 (25.3)	62.4 (22.2)	<0.001
**Gender (% men)**	50.3%	51.0%	50.5%	0.720
**Surgery during admission**				
**None**	69.3%	68.7%	67.4%	<0.001
**NHSN operative procedure**	17.9%	20.6%	17.5%	
**Non-NHSN operative procedure**	12.8%	10.7%	10.1%	
**McCabe score ^c^**				
**Non-fatal**	77.0%	74.6%	62.6%	<0.001
**Fatal**	12.2%	11.8%	11.6%	
**Rapidly fatal**	10.8%	13.6%	16.4%	
**Presence of urinary catheter**	33.5%	35.5%	41.2%	<0.001
**Presence of intubation**	3.4%	4.5%	5.6%	<0.001
**Presence of central venous catheter**	8.2%	9.9%	11.0%	<0.001
**Presence of peripheral venous catheter**	63.1%	67.6%	71.0%	<0.001

^a^ Based on the Chi-Squared test or on the ANOVA. ^b^ Results are reported as mean (standard deviation). ^c^ Classification of the severity of underlying medical conditions disregarding the influence of acute infections: non-fatal disease (expected survival at least five years); fatal disease (expected survival between one and five years); rapidly fatal disease (expected death within one year). Abbreviations: NHSN, National Healthcare Safety Network.

**Table 2:** Comparison of characteristics between infected and non-infected patients.

Characteristics	Univariate Analysis ^a^			Logistic Regression ^b^	
	Non-Infected	Infected	*p*-Value	OR	*p*-Value
**Age, years ^c^**	59.6 (24.3)	63.3 (22.1)	<0.001	1.00 (0.99–1.01)	0.491
**Gender (% men)**	50.5%	53.1%	0.111	0.96 (0.83–1.12)	0.606
**Surgery during admission**					
**None**	70.1%	59.9%	<0.001	Ref	
**Non-NHSN**	11.4%	13.1%		0.96 (0.76–1.21)	0.732
**NHSN**	18.5%	27.0%		1.04 (0.87–1.24)	0.682
**McCabe score ^d^**					
**Non-fatal**	74.7%	46.6%	<0.001	Ref	
**Fatal**	11.8%	24.1%		1.70 (1.39–2.07)	<0.001
**Rapidly fatal**	13.5%	29.3%		1.91 (1.57–2.31)	<0.001
**Presence of urinary catheter**	35.7%	61.2%	<0.001	1.16 (0.97–1.38)	0.101
**Presence of intubation**	3.7%	19.3%	<0.001	1.88 (1.49–2.38)	<0.001
**Presence of central venous catheter**	8.1%	38.8%	<0.001	3.21 (2.61–3.95)	<0.001
**Presence of peripheral venous catheter**	67.4%	69.9%	0.976	0.99 (0.82–1.21)	0.951
**Antibiotic use prevalence**	51.0%	96.6%	<0.001	18.87 (13.08–27.22)	<0.001

^a^ Based on the Student’s *t*-test or the Chi-Squared test. ^b^ Logistic regression model included all the variables reported in the table. ^c^ Results are reported as mean (standard deviation). ^d^ Classification of the severity of underlying medical conditions disregarding the influence of acute infections: non-fatal disease (expected survival at least five years); fatal disease (expected survival between one and five years); rapidly fatal disease (expected death within one year). Abbreviations: Ref, Reference group; NHSN, National Healthcare Safety Network.

**Table 3:** Indication for antimicrobial use by year of the Point Prevalence Survey.

Indication	2016 (n = 4271)	2017 (n = 4156)	2018 (n = 4414)	*p*-Value ^a^
**Surgical prophylaxis**	27.0%	26.7%	21.5%	<0.001
**Treatment of community infection**	22.3%	22.2%	24.5%	0.017
**Treatment of hospital infection**	9.9%	7.3%	10.1%	<0.001
**Treatment of infection acquired in LTCF**	1.5%	1.9%	1.6%	0.330
**Medical prophylaxis**	34.1%	35.6%	36.4%	0.073
**Other indication/Unknown**	5.2%	6.3%	5.8%	0.092

^a^ Based on the Chi-Squared test. Abbreviation: LTCF, Long-term care facility.

## Supplementary Materials

The following are available online at https://www.mdpi.com/2079-6382/10/1/1/s1, Figure S1: Relationship between prevalence of antibiotic use, prevalence of HAI and number of patients surveyed in each hospital. (A) The bubble graph reports the prevalence of HAI (*x*-axis) and the prevalence of antibiotic use (*y*-axis) in each hospital. The size of each bubble is proportional to the number of patients surveyed in each hospital. (B) This plot shows the correlation between the prevalence of HAI (*x*-axis) and the number of patients surveyed in each hospital (*y*-axis), Table S1: Distribution of the types of HAI by year of the Point Prevalence Survey, Table S2: Distribution of the antimicrobial agents by year of the Point Prevalence Survey.
