# Supplementary material for: Three-Year Trends of Healthcare-Associated Infections and Antibiotic Use in Acute Care Hospitals: Findings from 2016–2018 Point Prevalence Surveys in Sicily, Italy
Source: Antibiotics (Basel). 2020 Dec 22;10(1):1. doi: 10.3390/antibiotics10010001 (PMC7822200; doi:10.3390/antibiotics10010001)
Supplement: Supplementary file 1 [file antibiotics-10-00001-s001.pdf]

antibiotics

Supplementary Materials

Three-Year Trends of Healthcare-Associated Infections and Antibiotic Use in Acute Care Hospitals: Findings from 2016–2018 Point Prevalence Surveys in Sicily, Italy

Martina Barchitta [1], Andrea Maugeri [1], Maria Clara La Rosa [1], Claudia La Mastra [1], Giuseppe Murolo [2] and Antonella Agodi [1,3,*]

[1] Department of Medical and Surgical Sciences and Advanced Technologies "GF Ingrassia", University of Catania, 95123 Catania, Italy; martina.barchitta@unict.it (M.B.); andrea.maugeri@unict.it (A.M.); mariclalarosa@gmail.com (M.C.L.R.); claudia.lamastra@unict.it (C.L.M.)
[2] Regional Health Authority of the Sicilian Region, 90145 Palermo, Italy; giuseppe.murolo@regione.sicilia.it
[3] AOU Policlinico "G. Rodolico-San Marco", 95123 Catania, Italy
* Correspondence: agodia@unict.it

Index

- **Supplementary Table S1.** Distribution of the types of HAI by year of the Point Prevalence Survey
- **Supplementary Table S2.** Distribution of the antimicrobial agents by year of the Point Prevalence Survey
- **Supplementary Figure S1.** Relationship between prevalence of antibiotic use, prevalence of HAI and number of patients surveyed in each hospital.

Table S1. Distribution of the types of HAI by year of the Point Prevalence Survey.

Types of Infection	2016 (n = 349)	2017 (n = 317)	2018 (n = 371)
Urinary tract infections	20.3%	14.5%	21.8%
Pneumonia	28.5%	28.4%	31.5%
Surgical site infections	15.0%	14.2%	11.1%
Bloodstream	5.1%	6.6%	7.0%
Gastrointestinal system infections	4.0%	9.2%	4.9%
Skin and soft tissue infections	5.4%	9.2%	3.8%
Eye, ears, nose or mouth infections	1.1%	1.9%	1.6%
Systemic infections	3.4%	1.6%	1.3%
Cardiovascular system infections	2.0%	1.3%	1.6%
Central vascular catheter-related infections	3.1%	5.4%	4.9%
Peripheral vascular catheter-related infections	1.4%	0.9%	1.1%
Lower respiratory tract infections	5.4%	3.8%	5.9%
Bone and joint infections	2.8%	1.6%	1.9%
Reproductive tract infections	0.3%	0.3%	0.3%
Central nervous system infections	0.3%	0.3%	0.8%

Table S2. Distribution of the antimicrobial agents by year of the Point Prevalence Survey.

Antimicrobials	2016 (n = 4420)	2017 (n = 4394)	2018 (n = 4782)	*p*-Value
**Third-generation cephalosporins**	21.86%	21.78%	22.38%	0.752
**Fluoroquinolones**	19.00%	18.93%	14.37%	<0.001
**Combinations of penicillins, incl. betalactamase inhibitors**	15.00%	13.18%	14.62%	0.035
**Carbapenems**	7.51%	7.26%	7.49%	0.883
**Glycopeptide antibacterials**	6.31%	5.80%	5.00%	0.023
**First-generation cephalosporins**	5.75%	8.74%	7.80%	<0.001
**Imidazole derivatives**	4.59%	4.26%	4.33%	0.717
**Penicillins, extended spectrum**	3.51%	3.12%	3.85%	0.164
**Aminoglycosides**	2.74%	2.59%	2.68%	0.916
**Macrolides**	2.24%	2.12%	4.18%	<0.001

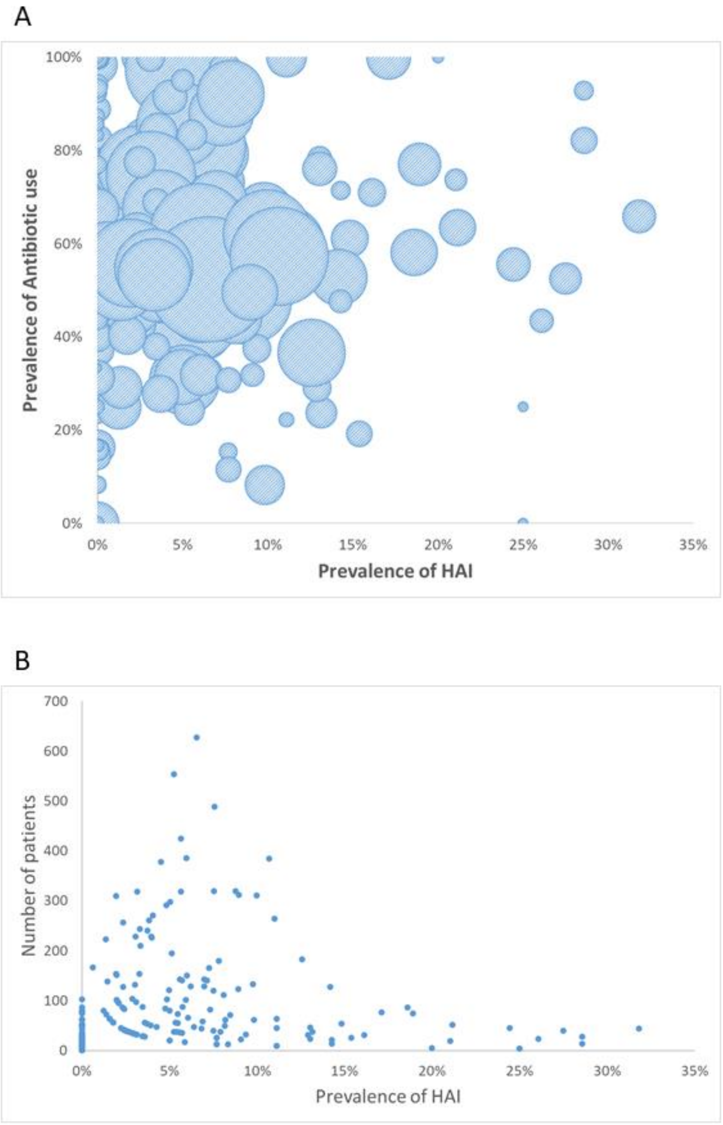

Figure S1. Relationship between prevalence of antibiotic use, prevalence of HAI and number of patients surveyed in each hospital. (**A**) The bubble graph reports the prevalence of HAI (x-axis) and the prevalence of antibiotic use (y-axis) in each hospital. The size of each bubble is proportional to the number of patients surveyed in each hospital. (**B**) This plot shows the correlation between the prevalence of HAI (x-axis) and the number of patients surveyed in each hospital (y-axis).
